# Low Prognostic Nutritional Index Correlates with Worse Survival in Patients with Advanced NSCLC following EGFR-TKIs

**DOI:** 10.1371/journal.pone.0147226

**Published:** 2016-01-19

**Authors:** Jin Sheng, Yun-Peng Yang, Yu-Xiang Ma, Tao Qin, Zhi-Huang Hu, Shao-Dong Hong, Ting Zhou, Yan Huang, Hong-Yun Zhao, Li Zhang

**Affiliations:** 1 Medical Oncology of Sun Yat-Sen University Cancer Center, Guangzhou, China; 2 State Key Laboratory of Oncology in South China, Guangzhou, China; 3 Collaborative Innovation Center for Cancer Medicine, Guangzhou, China; Univesity of Texas Southwestern Medical Center at Dallas, UNITED STATES

## Abstract

**Objective:**

This study was designed to demonstrate the prognostic value of prognostic nutritional index (PNI), a reflection systemic immunonutritional status, on the long-term survival of patients taking epidermal growth factor receptor (EGFR)-tyrosine kinase inhibitors (TKIs).

**Methods:**

In this retrospective study, eligible advanced NSCLC patients with sensitive EGFR mutations (exon 19 deletion or L858R in exon 21) were included to investigate the correlation between the PNI and overall survival (OS). The PNI was calculated as 10 x serum albumin value (g/dl) + 0.005 x peripheral lymphocyte count (per mm^3^). The prognostic significance of PNI and other clinicopathologic factors was identified by univariate and multivariate analysis.

**Results:**

Finally, 144 patients met the inclusion criteria. The optimal cut-off value of PNI for survival stratification was 48.78. Compared with high PNI group (n = 81), low PNI (n = 63) was significantly associated with elevated C-reactive protein (CRP) level and non-response to TKIs. Overall survival was superior in the high PNI group (HR, 0.44, p = 0.004), especially for patient with L858R (HR, 0.37, p = 0.009) rather than 19 deletion (HR, 0.69, p = 0.401). The independent prognostic value of PNI was validated by multivariate analysis.

**Conclusion:**

This pilot investigation demonstrated that low prognostic nutritional index correlates with worse survival for patients with advanced NSCLC and taking EGFR-TKIs. The assessment of a convenient index, known as PNI, worth attention in routine clinical practice for patients following EGFR-TKIs treatment.

## Introduction

Lung cancer remains the leading cause of cancer-related mortality [1]. Non-small-cell lung cancer (NSCLC) accounts for approximately 85% of lung cancers [2]. Compared with chemotherapy, molecular-targeted therapies, such as epidermal growth factor receptor (EGFR) inhibitors, have recently gained great attention for their potential to improve survival and quality of life with acceptable side effects [3–5] The presence of sensitive EGFR mutations is regarded as not only a predictive but also prognostic factor for the efficacy of EGFR-TKIs [6–8].

There is increasing evidence that the nutritional and immunological status is closely related to the long-term outcome of patients with malignancies [9, 10]. The prognostic nutritional index (PNI), which is calculated on the basis of serum albumin level and total lymphocyte count in peripheral blood, is originally employed as a reflection of pre-treatment immunonutritional status [11]. Recent studies have revealed that high PNI was a favorable prognostic factor in a variety of cancer types [12–15]. However, the prognostic value of PNI in patients treated with EGFR-TKIs is not yet fully evaluated. Moreover, EGFR exon 19 deletion and L858R are now distinguished as two different subgroups based on disparate sensitivity to EGFR-TKIs [16–18]. Whether the systemic inflammation interaction varies between two EGFR mutation types is also undefined. Therefore, in this retrospective study, we evaluated the association between pre-treatment PNI and clinicopathological factors as well as survival data for patients with EGFR sensitive mutations and treated with EGFR-TKIs.

## Materials and Methods

### Participant Identification

We retrospectively reviewed all patients who were histologically diagnosed as advanced NSCLC between Jan 2011 and December 2013 in Sun Yat-Sen University Cancer Center (SYSUCC, Guangzhou, China). Detailed inclusion criteria were as follows: (1) ≥18 years old. (2) With advanced (stage IIIB or IV) determined by the 7th edition of tumor, node, metastasis classification. (3) With EGFR sensitive mutations (including exon 19 deletion and L858R in exon 21) identified by fluorescent quantitative polymerase chain reaction (PCR) or amplification refractory mutation system (ARMS) and ever received EGFR-TKIs (gefitinib or erlotinib). (4) Available pre-treatment laboratory data. (5) Complete clinicopathological information. (6) Eastern Cooperative Oncology Group (ECOG) performance status of ≤2. Patients with infection fever, autoimmune diseases, previous or coexisting cancers other than NSCLC, or hematological disorders were excluded. The propensity score was used to minimize the potential selection bias. Patients with confirmed EGFR mutations received either gefitinib 250 mg/d or erlotinib 150 mg/d orally.

The study protocol was approved by the institutional review board of Sun Yat-Sen University Cancer Center (SYSUCC, Guangzhou, China) and written informed consent was obtained for each participant. The investigation have been conducted according to the principles expressed in the Declaration of Helsinki.

### Clinical Data Collection

The demographics and clinicopathlogical information such as age, gender, smoking status, stage, mutation type, line of EGFR-TKIs treatment and types of agent were retrospectively collected from electronic medical record system. Smokers were defined as those who had more than 100 lifetime cigarettes, including current and previous smokers. Pre-treatment blood parameters, serum albumin, CRP level were also obtained before (less than 1 week) the oral administration of EGFR-TKIs. PNI was then calculated with the following formula as previously described: 10 × serum albumin value (g/dl) + 0.005 × peripheral lymphocyte count (per mm^3^) [11]. This study followed the items that should be included in reports of observational studies as S1 STROBE Statement.

### Statistical Analysis

The OS was defined as the interval from the date of initiation of EGFR-TKIs to the date of death from any cause or last follow-up by telephone calls. The tumor responses were evaluated according to the Response Evaluation Criteria in Solid Tumors (RECIST 1.0). Response was defined as the disappearance of all target lesions (complete response) or partial response-at least a 30% decrease in the sum of the longest diameter of target lesions, taking as reference the baseline sum longest diameter. All patients were followed up to May 31th 2015 or death from any cause. Categorical variables were presented as the number of patients and percentages and were compared using Chi-square or Fisher’s exact test. Continuous variables were expressed as medians and ranges and were categorized at the median value. The optimal cut-off value of PNI was determined by an R software-engineered, web-based system (http://molpath.charite.de/cutoff/) [19]. Survival analysis were performed with SPSS version 22.0 (SPSS Inc., Chicago, IL, USA). Kaplan–Meier methodology was employed for survival analysis. Significant variables related to OS were further tested by multivariate analysis with Cox proportional hazards model. A two-sided p value of less than 0.05 was defined as significant.

## Results

### Patient Characteristics

**Fig 1:**
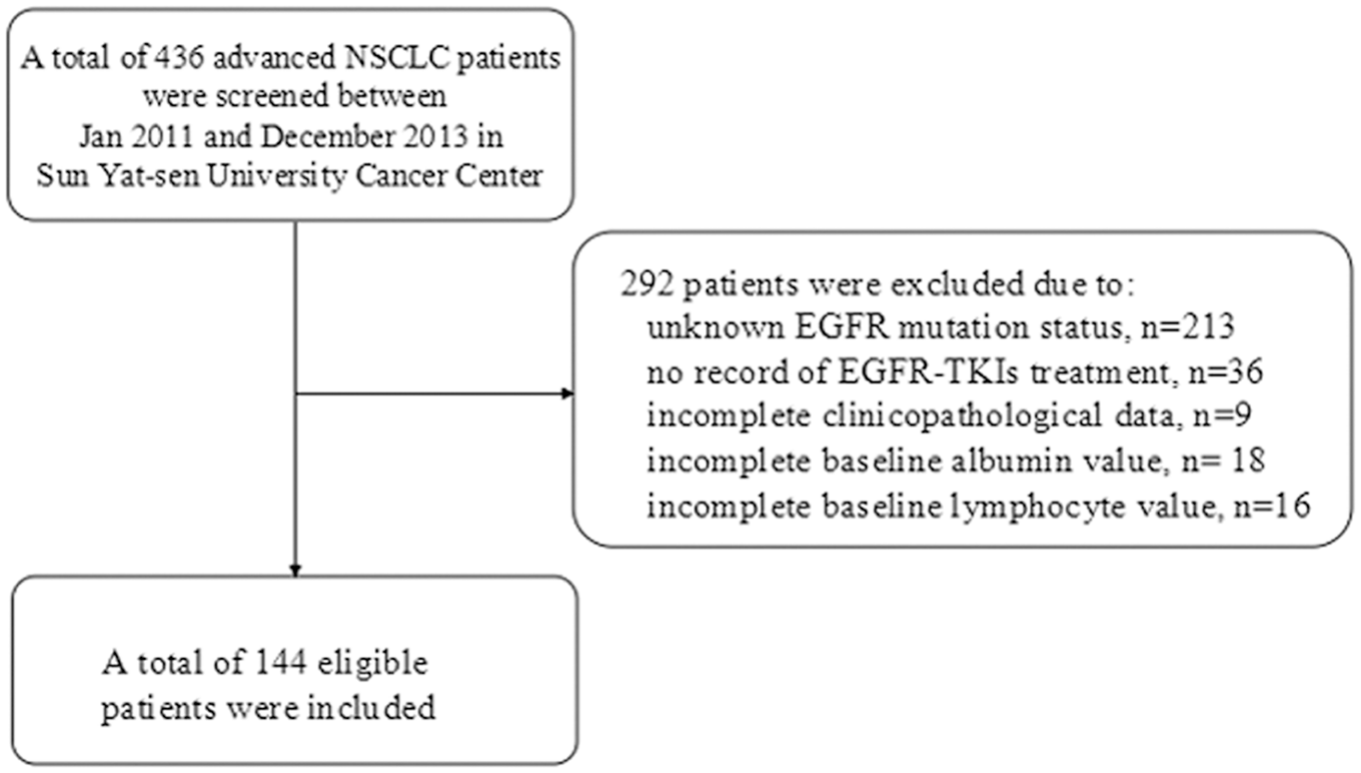
Flow chart of patients’ selection.

A total of 436 advanced NSCLC patients diagnosed in Sun Yat-sen University Cancer Center were screened between Jan 2011 and December 2013. One hundred and seventy three patients with EGFR-activating mutations were further screened for eligibility. 29 patients were excluded due to unavailable laboratory data. Finally, 144 NSCLC patients with sensitive EGFR mutations and treated with TKIs as first-line or maintenance treatment were included for analysis. The details of patients’ selection process are shown in Fig 1.

The baseline characteristics of eligible patients are shown in Table 1. The median age of the study population was 58 years (range: 25–81 years), and approximately half of the patients were males (n = 75, 52.1%). The majority of the patients were never smokers (n = 105, 72.9%). For cancer stage, 118 (81.9%) patients were initially diagnosed as metastatic NSCLC. There were 71 (49.3%) patients received EGFR-TKIs as first-line and 73 (50.7%) as maintenance strategy. The most commonly used EGFR-TKI was gefitinib (n = 111, 77.1%). As for the mutation type, 67 (46.5%) patients were detected with exon 19 deletion and 77 (53.5%) were L858R. The median value of CRP were 3.68 (range 0.23–134.17). Therefore, 78 patients (54.2%) were assigned to the relatively CRP-low group. 89 (61.8%) patients presented response to treatment, while 53 remained disease stable. Two patients underwent disease progression as the best response.

**Table 1:** Baseline characteristics stratified by pretreatment PNI level.

Characteristics	N(%)	PNI-low, N(%)	PNI-high, N(%)	OR (95%CI)^a^	*P* value
Total	144 (100%)	63 (43.7)	81 (56.3)		
Ages,years					
<58	81 (56.3)	36 (57.1)	45 (55.6)	1(Referent)	
≥58	63 (43.7)	27 (42.9)	36 (44.4)	1.07(0.55–2.07)	0.85
Gender					
Female	69 (47.9)	28 (44.4)	41 (50.6)	1(Referent)	
Male	75 (52.1)	35 (55.6)	40 (49.4)	0.78(0.40–1.51)	0.46
Line					
Maintenance	73 (50.7)	28 (44.4)	45 (55.6)	1(Referent)	
First-line	71 (49.3)	35 (55.6)	36 (44.4)	0.64(0.33–1.24)	0.19
Smoking					
Never smoker	105 (72.9)	43 (68.3)	62 (76.5)	1(Referent)	
Current or ex-smoker	39 (27.1)	20 (31.7)	19 (23.5)	0.66(0.31–1.38)	0.27
Stage					
IIIB	26 (18.1)	11 (17.5)	15 (18.5)	1(Referent)	
IV	118 (81.9)	52 (82.5)	66 (81.5)	0.93(0.39–2.19)	0.87
ECOG-PS					
0	41 (28.5)	19 (30.2)	22 (27.2)	1(Referent)	
≥1	103 (71.5)	44 (69.8)	59 (72.8)	0.92 (0.48–1.76)	0.81
EGFR mutation types					
L858R	77 (53.5)	39 (61.9)	38 (46.9)	1(Referent)	
19 deletion	67 (46.5)	24 (38.1)	43 (53.1)	1.84 (0.94–3.59)	0.07
Drug					
Erlotinib	33 (22.9)	12 (19.0)	21 (25.9)	1(Referent)	
Gefitinib	111 (77.1)	51 (81.0)	60 (74.1)	0.67(0.30–1.50)	0.33
CRP at baseline					
Relatively low group^b^	78 (54.2)	21 (33.3)	57 (70.4)	1(Referent)	
Relatively high group	66 (45.8)	42 (66.7)	24 (29.6)	0.21(0.10–0.43)	<0.0001
Response to TKIs^c^					
Response	89 (61.8)	30 (47.6)	59 (72.8)	1(Referent)	
Non-response	55 (38.2)	33 (52.4)	22 (27.2)	0.34(0.17–0.68)	0.002

NOTE:

^a^ Denoted as the potential of having high PNI between the given groups;

^b^ Classified according to the median value.

^c^ Evaluated according to RECIST guideline (Version 1.0.). Abbreviations: OR, odd ratio; CI, confidence interval; PS, performance status; CRP, C-reactive protein; PNI, prognostic nutritional index.

### Cut-Off Determination of PNI

**Fig 2:**
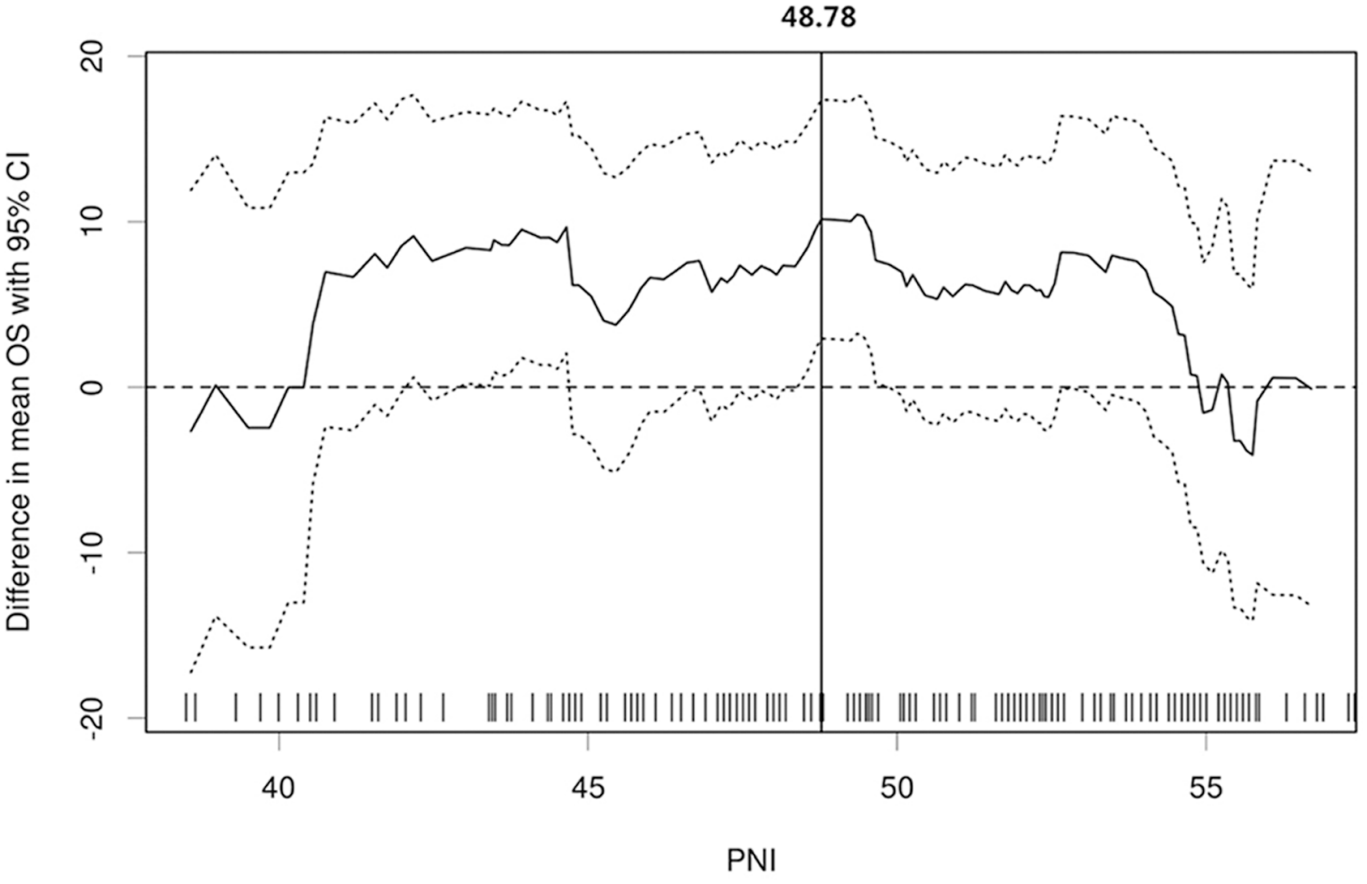
The optimal cut-off point plots generated by the biostatistical tool, *Cut-off Finder*. The vertical line designates the optimal cut-off point with the most significant *(log-rank test)* split.

The median value of PNI was 49.50 (range, 27.60–85.90). The optimal cut-off points of PNI for the stratification of OS was determined to be 48.78 (Fig 2). Based on this cut-off value, 81 (56.3%) patients were categorized as PNI-high group while the remaining 63 (43.7%) patients as PNI-low group.

### Association between Clinicopathological Variables and PNI

Clinical and laboratory factors stratified by PNI levels were presented in Table 1. In general, gender, age, smoking status, line of treatment and regimens were similar between the two groups. High PNI was significantly associated with relatively low CRP (p<0.0001) and those presented response to TKIs (p = 0.002). Besides, patients with exon 19 deletion tended to have high PNI though no significant difference was reached (p = 0.070).

### Association of PNI with OS

The median follow-up time was 32.0 months (range: 1.05–45.13 months). The ratio for loss to follow-up was 8.33% (n = 12). In general, the median OS were 31.2 months (95% CI: 26.2–36.3 months) while the median PFS was 13.4 months (95% CI: 10.1–16.7 months). Univariate analysis suggested that high PNI is significantly associated with longer OS compared with low PNI group (median OS in high vs low PNI group, 35.1 vs 25.7 months; HR, 0.44; 95% CI, 0.25–0.77; p = 0.004), yielding a significant reduction in the mortality risk of 56%. As shown in Table 2, other variables significantly associated with OS including ECOG (HR, 4.01; p <0.001), smoking status (HR, 1.99; p = 0.017), staging (HR, 2.33; p = 0.027) and line of treatment (HR, 1.98; p = 0.020). However, no significant difference in OS was detected regarding age (p = 0.556), gender (p = 0.637), CRP at baseline (p = 0.204), and regimens (p = 0.593). In terms of EGFR mutation type, exon 19 deletion was associated with modest but not significant survival superiority compared with L858R (33.5 vs 28.8 months, HR, 0.59; 95% CI, 0.34–1.03; p = 0.063). PNI was further validated by multivariate analysis as an independent prognostic factor. Patients with high PNI had 51% decrease in the risk of death compared with those with low PNI (HR, 0.41; 95%CI, 0.28–0.85; p = 0.002). Non-smokers, patient with local advanced stage and good PS were also independently predictors for better OS. S1 Fig. represented the over survival curves illustrating these independent prognostic factors.

**Table 2:** Univariate and multivariate analysis of clinicopathological parameters for the prediction of OS in patients with NSCLC.

Parameters	Median OS (95%CI)	Univariate analysis		Multivariate analysis	
		HR (95%CI)	*P*	HR(95%CI)	*P* value
Ages,years					
<58	33.5 (28.5–38.5)	1.00	-		
≥58	28.2 (19.5–36.9)	1.18 (0.68–2.03)	0.556		
Gender					
Female	33.5 (28.9–38.1)	1.00	-		
Male	31.1 (21.8–40.4)	1.14 (0.66–1.97)	0.637		
Line					
First-line	28.2 (23.0–33.4)	1.00	-	1.00	-
Maintenance	38.3 (26.8–49.8)	1.98 (1.11–3.51)	0.020	1.44 (0.77–2.70)	0.260
Smoking					
Never smoker	33.5 (29.2–37.8)	1.00	-	1.00	-
Current or ex-smoker	25.7 (15.2–36.1)	1.99 (1.13–3.51)	0.017	2.41 (1.32–4.41)	0.004
Stage					
IIIB	38.3 (27.5–49.1)	1.00	-	1.00	-
IV	28.8 (23.9–33.7)	2.33(1.10–4.93)	0.027	2.96 (1.29–6.79)	0.010
ECOG-PS					
0	38.3 (29.1–47.5)	1.00)	-	1.00	-
≥1	23.8 (17.8–29.8)	4.01 (2.08–7.73)	<0.001	6.33 (2.98–13.47)	<0.001
EGFR mutation types					
L858R	28.8 (21.9–35.8)	1.00	-		
Exon 19 deletion	33.5 (27.6–39.4)	0.59 (0.34–1.03)	0.063		
PNI at baseline					
PNI < 48.78	25.7 (19.3–32.0)	1.00	-	1.00	-
PNI ≥ 48.78	35.1 (NA)	0.44 (0.25–0.77)	0.004	0.41 (0.23–0.73)	0.002
Drug					
Gefitinib	31.2 (26.4–36.2)	1.00	-		
Erlotinib	28.8 (20.3–37.3)	1.20 (0.62–2.34)	0.593		
CRP at baseline					
Relatively low group	35.1 (30.8–39.4)	1.00	-		
Relatively high group	27.7 (24.2–31.3)	1.43(0.83–2.47)	0.204		

Abbreviations: OS, overall survival; CI, confidence interval; HR, hazard ratio; PS, performance status; CRP, C-reactive protein;PNI, prognostic nutritional index.

**Fig 3:**
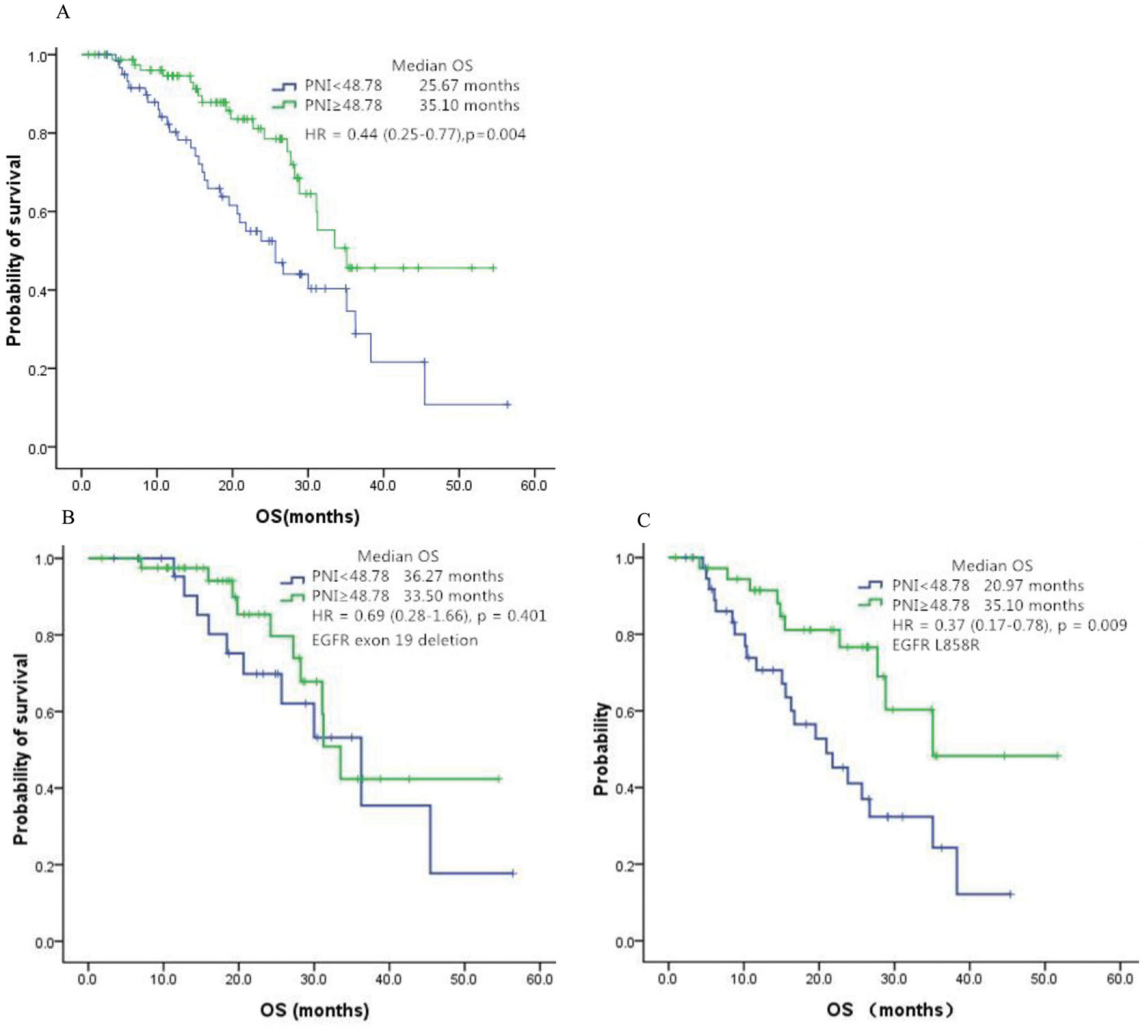
The prognostic value of PNI on overall survival curves in patients with EGFR exon 19 deletion or L858R. (**A)** Comparison of OS on patients with high PNI vs low PNI. (**B)** Comparison of high PNI vs low PNI in patients with EGFR exon 19 deletion. (**C)** Comparison of high PNI vs low PNI in patients with EGFR L858R.

**Fig 4:**
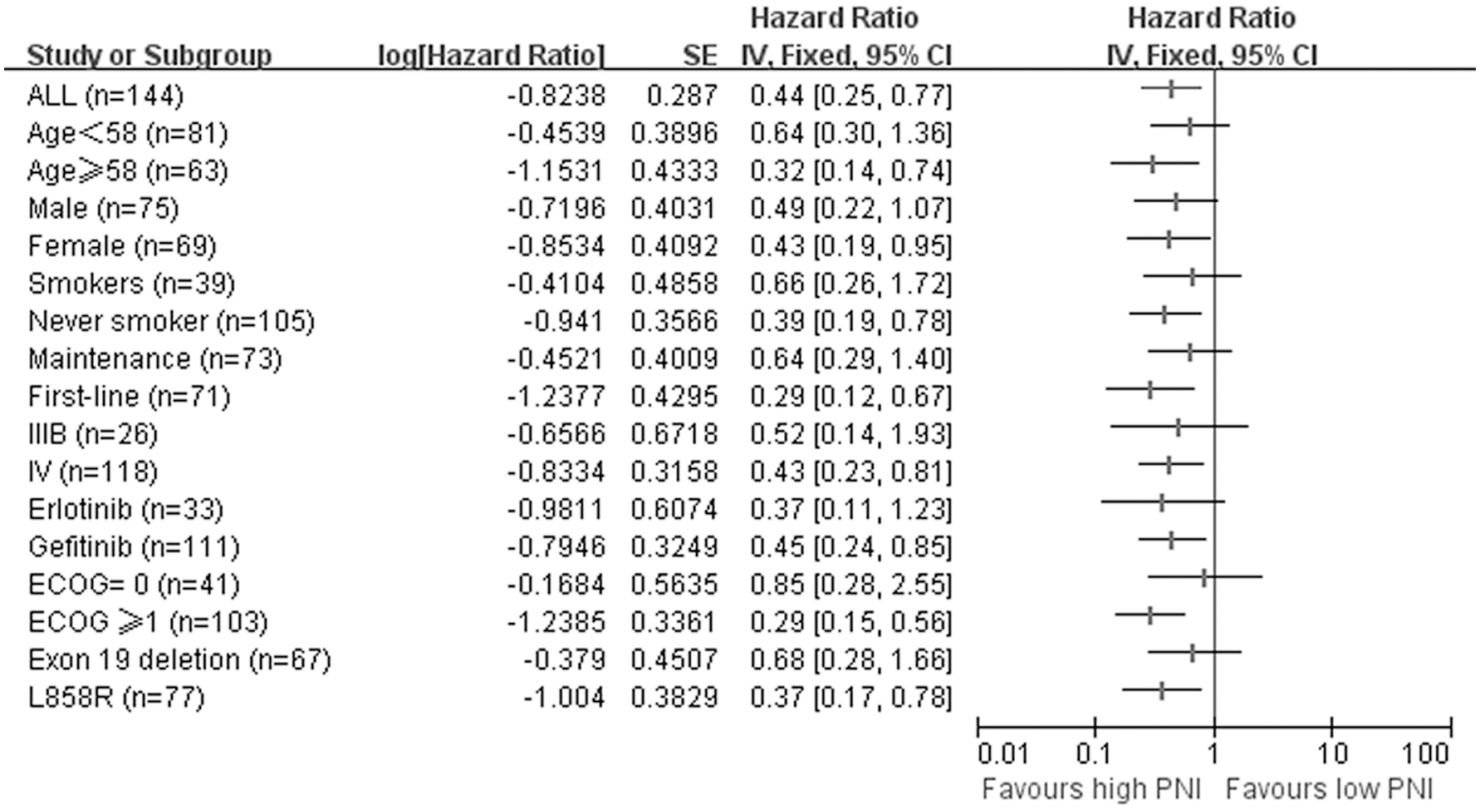
Forest plot for subgroup analysis of overall survival. Survival is for high PNI vs low PNI. Data are derived from Cox’s analysis without covariates.

In order to investigate the consistency of PNI as a prognostic factor for patients with advanced NSCLC receiving EGFR-TKIs, we performed subgroup analysis according to baseline characteristics. The prognostic value of PNI on overall survival curves was presented in Fig 3A. Of note, for patient harboring L858R mutations, the survival benefit of high PNI was more apparent (HR, 0.37; 95% CI, 0.17–0.78; p = 0.009, Fig 3C. The survival superiority was not significant in those have EGFR exon 19 deletion (HR, 0.69; 95% CI, 0.28–1.66; p = 0.401, Fig 3B). Higher PNI significantly predicted favorable OS in patients older than 58, female and never smokers. PNI was also a significant prognostic factor in first-line setting and stage IV. Besides, high PNI correlated with superior OS in patients with ECOG-PS ≥1 and treated with gefitinib. However, for patients less than 58, good performance status, males, stage IIIB, current or ex-smokers and treated with erlotinib, higher PNI was only correlated with non-significant OS superiority. The result were summarized in Fig 4.

## Discussion

Recent data have suggested that the systemic inflammatory response plays an important role in the development and progression of cancer. It was reported that induction of inflammation was sufficient to decrease the tumor response to erlotinib [16]. Various laboratory inflammation markers have been linked to the prognosis of advanced NSCLC, such as CRP, mGPS and neutrophil to lymphocyte ratio (NLR) [20–22]. Based on systemic albumin and peripheral lymphocyte count, the PNI was initially employed to assess the immunological and nutritional status of patients undergoing gastrointestinal surgery [19, 23]. Recently, PNI has been widely accepted as an indicator of nutritional and inflammatory status and correlates with efficacy and long-term outcomes of various malignances [24, 25].

To the best of our knowledge, this is the first report about the predictive value of PNI on OS in NSCLC patients harboring EGFR mutations and following EGFR-TKIs. Our findings revealed that low pre-treatment PNI level correlated to worse OS of advanced NSCLC patients taking EGFR-TKIs. Our results were in line with previously published studies demonstrating the significant relationship between PNI and prognosis for cancers. High PNI was significantly associated with preferable overall survival in colorectal cancer, hepatocellular carcinoma and triple-negative breast cancer [26–28]. Yao et al reported that PNI is an independent prognostic factor in malignant pleural mesothelioma. Patients with lower PNIs (PNI < 44.6) had greater risk of death than those with higher PNIs (PNI≥ 44.6; HR, 2.290; 95% CI: 1.42–3.71; p = 0.001) [29].

Malnutrition is partially reflected by hypoalbuminemia and has intensively been studied as a significant prognostic factor in cancer patients, suggesting the unfavorable role of immunonutritional status on the clinical outcome of malignancies, including NSCLC [30]. Reduced lymphocyte reveals the impairment of innate cellular immunity and correlates to disease severity and poor prognosis [31]. Taken together, the PNI based on serum albumin and lymphocyte is of prognostic significance as reflectors of systemic immunity and nutritional status and serve as a rational significant prognostic factor in NSCLC. In this study, as we present in Table 1, high PNI was significantly associated with relatively low CRP (p<0.0001) and those presented response to TKIs (p = 0.002). This may serve as one explanation for why high-PNI level correlates with better overall survival, since it was negatively related to host inflammation level but positively associated with EGFR-TKI efficacy.

Other factors such as the presence of sensitive EGFR mutations [6, 7], baseline CRP level [21], metastasis [32] and smoking status [33] have been identified as significant prognostic factors for the efficacy of EGFR-TKIs. Our result analogously revealed that EGFR-TKIs as maintenance regimens, stage IIIB and never smokers were independently predictors for better OS. Although it is strongly recommended that EGFR-TKIs should be used for patient with known EGFR-activating mutations, there is no head-to head comparison of first-line application of TKIs with maintenance application after 4–6 cycles of front platinum-doublet chemotherapy. Therefore, whether the long-term efficacy of TKIs is superior as maintenance regimen than first-line application remains prospective evaluation.

The optimal method to determine the cutoff value of PNI has not reached consensus. Median or mean value and the receiver operating characteristic (ROC) curve analysis are widely used methods [28, 29, 34]. The optimal cut-off value of PNI in the present study was determined by an R software-engineered, web-based system, which provides robust and reasonable result when discriminating time-to-event end points like OS [15].

The association between clinicopathological variables and PNI was further explored to exclude potential bias. Briefly, higher PNI correlated to significant OS superiority in older patients, female and never smokers. PNI was also a significant prognostic factor in first-line setting and metastatic stage. Moreover, high PNI was associated with better OS in those harboring L858R mutations and treated with gefitinib. However, for patients less than 58, males, stage IIIB, current or ex-smokers, EGFR exon 19 deletion or treated with erlotinib, the survival benefit of higher PNI was not significant. One explanation is that the enrolled number of patients who was current or ex-smokers, stage IIIB or treated with erlotinib was relatively smaller and insufficient to reach a significant result. Another interpretation is that high PNI was a meaningful prognostic factor especially for those suffering unpleasant prognostic factor, such as elder people, metastasis and with L858R mutation. Of note, inflammation plays a vital role in metastasis and resistance to molecular targeted therapy in lung cancer [16]. Patients with exon 19 deletion present longer progression-free survival, compared with those harboring L858R [18]. Our finding illustrate a potential systemic inflammation-involved mechanism for this discrepancy. Further studies on independent multicenter cohorts are warranted to validate our findings.

Our study was limited by its retrospective nature and relatively small number of patients from one single center. Therefore, it could be considered as a pilot investigation from which a prospective study could be designed and carried out to verify our finding. Besides, among the eligible patients, there was no application of EGFR-TKIs as second-line therapy, which imposes restrictions on the interpretation of our result. Moreover, the impact of EGFR-TKIs on PNI and this significance tailoring prognosis was hard to be evaluated. However, as a whole, we demonstrated that the baseline PNI level correlated with the OS in patients harboring EGFR sensitive mutations and following EGFR-TKIs treatment. Besides, our study pointed out a potential intervention pointcut through which anti-inflammation and nutrition support may improve the prognosis.

## Conclusion

This pilot investigation demonstrated that low prognostic nutritional index correlates with worse survival for patients with advanced NSCLC and taking EGFR-TKIs. The assessment of a convenient index, known as PNI, worth attention in routine clinical practice for patients following EGFR-TKIs treatment.

## Supporting Information

S1 Fig Overall survival curves illustrating other independent prognostic factors. (A) Current or ex-smokers vs never smokers. (B) Stage IV vs IIIB. C. ECOG-PS≥1 vs ECOG-PS = 0.
(DOC)

S1 STROBE Statement A checklist of items that should be included in reports of observational studies. (DOCX)
