# Supplementary material for: Low Prognostic Nutritional Index Correlates with Worse Survival in Patients with Advanced NSCLC following EGFR-TKIs
Source: PLoS One. 2016 Jan 19;11(1):e0147226. doi: 10.1371/journal.pone.0147226 (PMC4718699; doi:10.1371/journal.pone.0147226)

**S1 Figure. Overall survival curves illustrating other independent prognostic factors.** (A) Current or ex-smokers vs never smokers. (B) Stage IV vs IIIB. C. ECOG-PS≥1 vs ECOG-PS=0.


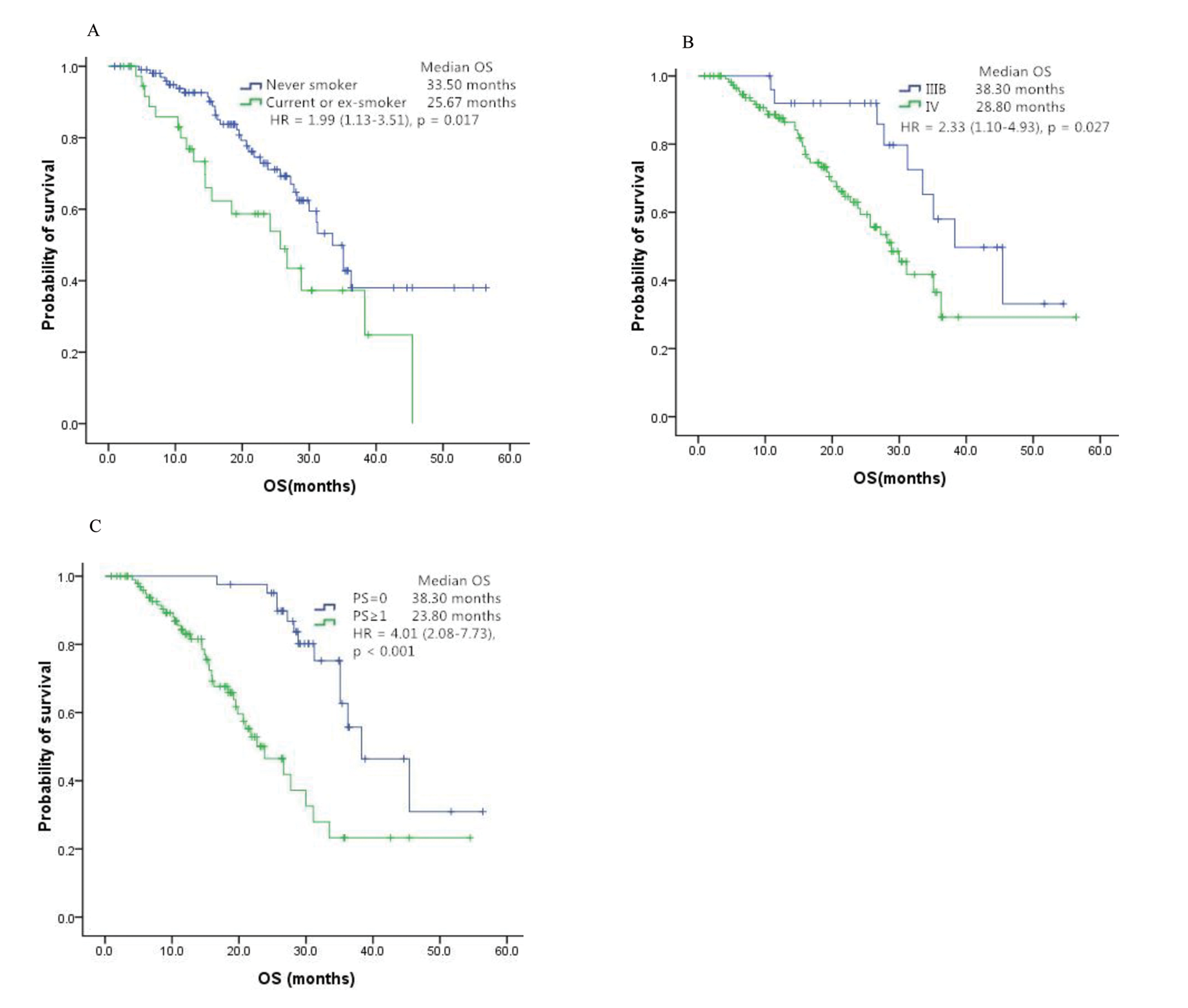

Supplement: S1 Fig — (A) Current or ex-smokers vs never smokers. (B) Stage IV vs IIIB. C. ECOG-PS≥1 vs ECOG-PS = 0. (DOC) [file pone.0147226.s001.doc]
