# Supplementary material for: Low Prognostic Nutritional Index Correlates with Worse Survival in Patients with Advanced NSCLC following EGFR-TKIs
Source: PLoS One. 2016 Jan 19;11(1):e0147226. doi: 10.1371/journal.pone.0147226 (PMC4718699; doi:10.1371/journal.pone.0147226)
Supplement: S1 STROBE Statement — (DOCX) [file pone.0147226.s002.docx]

**STROBE Statement—checklist of items that should be included in reports of observational studies**

|  | Item No. | Recommendation | Page  No. | Relevant text from manuscript |
| --- | --- | --- | --- | --- |
| **Title and abstract** | 1 | (*a*) Indicate the study’s design with a commonly used term in the title or the abstract | 2 | This study was designed to demonstrate the prognostic value of prognostic nutritional index (PNI), a reflection systemic immunonutritional status, on the long-term survival of patients taking epidermal growth factor receptor (EGFR)-tyrosine kinase inhibitors (TKIs). |
|  |  | (*b*) Provide in the abstract an informative and balanced summary of what was done and what was found | 2 | In this retrospective study, eligible advanced NSCLC patients with sensitive EGFR mutations (exon 19 deletion or L858R in exon 21) were included to investigate the correlation between the PNI and overall survival (OS). The PNI was calculated as 10 x serum albumin value (g/dl) + 0.005 x peripheral lymphocyte count (per mm3). The prognostic significance of PNI and other clinicopathologic factors was identified by univariate and multivariate analysis. 144 patients met the inclusion criteria. The optimal cut-off value of PNI for survival stratification was 48.78. Compared with high PNI group (n=81), low PNI (n = 63) was significantly associated with elevated C-reactive protein (CRP) level and non-response to TKIs. Overall survival was superior in the high PNI group (HR, 0.44, p = 0.004), especially for patient with L858R (HR, 0.37, p = 0.009 ) rather than 19 deletion (HR, 0.69, p = 0.401). The independent prognostic value of PNI was validated by multivariate analysis. |
| Introduction | | | |  |
| Background/rationale | 2 | Explain the scientific background and rationale for the investigation being reported | 3 | Recent studies have revealed that high PNI was a favorable prognostic factor in a variety of cancer types [12-15]. However, the prognostic value of PNI in patients treated with EGFR-TKIs is not yet fully evaluated. Moreover, EGFR exon 19 deletion and L858R are now distinguished as two different subgroups based on disparate sensitivity to EGFR-TKIs [16-18]. Whether the systemic inflammation interaction varies between two EGFR mutation types is also undefined. |
| Objectives | 3 | State specific objectives, including any prespecified hypotheses | 3 | Therefore, in this retrospective study, we evaluated the association between pretreatment PNI and clinicopathological factors as well as survival data for patients with EGFR sensitive mutations and treated with EGFR-TKIs. |
| Methods | | | |  |
| Study design | 4 | Present key elements of study design early in the paper | 4 | Retrospective study |
| Setting | 5 | Describe the setting, locations, and relevant dates, including periods of recruitment, exposure, follow-up, and data collection | 4-5 | We retrospectively reviewed all patients who were histologically diagnosed as advanced NSCLC between Jan 2011 and December 2013 in Sun Yat-Sen University Cancer Center (SYSUCC, Guangzhou, China). Patients with confirmed EGFR mutations received either gefitinib 250 mg/d or erlotinib 150 mg/d orally. The tumor responses were evaluated according to the Response Evaluation Criteria in Solid Tumors (RECIST 1.0). All patients were followed up to May 31th 2015 or death from any cause. |
| Participants | 6 | (*a*) *Cohort study*—Give the eligibility criteria, and the sources and methods of selection of participants. Describe methods of follow-up  *Case-control study*—Give the eligibility criteria, and the sources and methods of case ascertainment and control selection. Give the rationale for the choice of cases and controls  *Cross-sectional study*—Give the eligibility criteria, and the sources and methods of selection of participants | 4 | Detailed inclusion criteria were as follows: (1) ≥18 years old. (2) With advanced (stage IIIB or IV) determined by the 7th edition of tumor, node, metastasis classification. (3) With EGFR sensitive mutations (including exon 19 deletion and L858R in exon 21) identified by fluorescent quantitative polymerase chain reaction (PCR) or amplification refractory mutation system (ARMS) and ever received EGFR-TKIs (gefitinib or erlotinib). (4) Available pre-treatment laboratory data. (5) Complete clinicopathological information. (6) Eastern Cooperative Oncology Group (ECOG) performance status of ≤2. Patients with infection fever, autoimmune diseases, previous or coexisting cancers other than NSCLC, or hematological disorders were excluded. |
|  |  | (*b*) *Cohort study*—For matched studies, give matching criteria and number of exposed and unexposed  *Case-control study*—For matched studies, give matching criteria and the number of controls per case | 4 | The propensity score was used to minimize the potential selection bias. |
| Variables | 7 | Clearly define all outcomes, exposures, predictors, potential confounders, and effect modifiers. Give diagnostic criteria, if applicable | 5 | PNI was then calculated with the following formula as previously described: 10 × serum albumin value (g/dl) + 0.005 × peripheral lymphocyte count (per mm3) [11].This study followed the items that should be included in reports of observational studies as S1 STROBE Statement. The OS was defined as the interval from the date of initiation of EGFR-TKIs to the date of death from any cause or last follow-up by telephone calls. |
| Data sources/ measurement | 8* | For each variable of interest, give sources of data and details of methods of assessment (measurement). Describe comparability of assessment methods if there is more than one group | 5 | Categorical variables were presented as the number of patients and percentages and were compared using Chi-square or Fisher’s exact test. Continuous variables were expressed as medians and ranges and were categorized at the median value. The optimal cut-off value of PNI was determined by an R software-engineered, web-based system (http://molpath.charite.de/cutoff/) [19]. Survival analysis were performed with SPSS version 22.0 (SPSS Inc., Chicago, IL, USA). Kaplan–Meier methodology was employed for survival analysis. Significant variables related to OS were further tested by multivariate analysis with Cox proportional hazards model. A two-sided p value of less than 0.05 was defined as significant. |
| Bias | 9 | Describe any efforts to address potential sources of bias | 4 | The propensity score was used to minimize the potential selection bias. |
| Study size | 10 | Explain how the study size was arrived at | 5-6 | A total of 436 advanced NSCLC patients diagnosed in Sun Yat-sen University Cancer Center were screened between Jan 2011 and December 2013. One hundred and seventy three patients with EGFR-activating mutations were further screened for eligibility. 29 patients were excluded due to unavailable laboratory data. Finally, 144 NSCLC patients with sensitive EGFR mutations and treated with TKIs as first-line or maintenance treatment were included for analysis. |

Continued on next page

| Quantitative variables | 11 | Explain how quantitative variables were handled in the analyses. If applicable, describe which groupings were chosen and why | 5 | Continuous variables were expressed as medians and ranges and were categorized at the median value. Categorical variables were presented as the number of patients and percentages and were compared using Chi-square or Fisher’s exact test.The optimal cut-off value of PNI was determined by an R software-engineered, web-based system (http://molpath.charite.de/cutoff/) [19] |
| --- | --- | --- | --- | --- |
| Statistical methods | 12 | (*a*) Describe all statistical methods, including those used to control for confounding | 5 | Chi-square or Fisher’s exact test, survival analysis. |
|  |  | (*b*) Describe any methods used to examine subgroups and interactions | 5 | Categorical variables were presented as the number of patients and percentages and were compared using Chi-square or Fisher’s exact test |
|  |  | (*c*) Explain how missing data were addressed | N/A |  |
|  |  | (*d*) *Cohort study*—If applicable, explain how loss to follow-up was addressed  *Case-control study*—If applicable, explain how matching of cases and controls was addressed  *Cross-sectional study*—If applicable, describe analytical methods taking account of sampling strategy | 5 | The OS was defined as the interval from the date of initiation of EGFR-TKIs to the date of death from any cause or last follow-up by telephone calls. The tumor responses were evaluated according to the Response Evaluation Criteria in Solid Tumors (RECIST 1.0). All patients were followed up to May 31th 2015 or death from any cause. |
|  |  | (*e*) Describe any sensitivity analyses | N/A |  |
| Results | | | | |
| Participants | 13* | (a) Report numbers of individuals at each stage of study—eg numbers potentially eligible, examined for eligibility, confirmed eligible, included in the study, completing follow-up, and analysed | 6 | A total of 436 advanced NSCLC patients diagnosed in Sun Yat-sen University Cancer Center were screened between Jan 2011 and December 2013. One hundred and seventy three patients with EGFR-activating mutations were further screened for eligibility. 29 patients were excluded due to unavailable laboratory data. Finally, 144 NSCLC patients with sensitive EGFR mutations and treated with TKIs as first-line or maintenance treatment were included for analysis. The details of patients’ selection process are shown in Fig 1. |
|  |  | (b) Give reasons for non-participation at each stage | 6 |  |
|  |  | (c) Consider use of a flow diagram | 6 |  |
| Descriptive data | 14* | (a) Give characteristics of study participants (eg demographic, clinical, social) and information on exposures and potential confounders | 6 | The baseline characteristics of eligible patients are shown in Table 1. |
|  |  | (b) Indicate number of participants with missing data for each variable of interest | N/A |  |
|  |  | (c) *Cohort study*—Summarise follow-up time (eg, average and total amount) | 7 | The median follow-up time was 32.0 months (range: 1.05-45.13 months). The ratio for loss to follow-up was 8.33% (n = 12). |
| Outcome data | 15* | *Cohort study*—Report numbers of outcome events or summary measures over time | 6 ,7 |  |
|  |  | *Case-control study—*Report numbers in each exposure category, or summary measures of exposure | N/A |  |
|  |  | *Cross-sectional study—*Report numbers of outcome events or summary measures | N/A |  |
| Main results | 16 | (*a*) Give unadjusted estimates and, if applicable, confounder-adjusted estimates and their precision (eg, 95% confidence interval). Make clear which confounders were adjusted for and why they were included | 7,8 | Association of PNI with OS. |
|  |  | (*b*) Report category boundaries when continuous variables were categorized | 6 | The median value of PNI was 49.50 (range, 27.60–85.90). The optimal cut-off points of PNI for the stratification of OS was determined to be 48.78 (Fig 2). |
|  |  | (*c*) If relevant, consider translating estimates of relative risk into absolute risk for a meaningful time period | N/A |  |

Continued on next page

| Other analyses | 17 | Report other analyses done—eg analyses of subgroups and interactions, and sensitivity analyses | 8 | In order to investigate the consistency of PNI as a prognostic factor for patients with advanced NSCLC receiving EGFR-TKIs, we performed subgroup analysis according to baseline characteristics. |
| --- | --- | --- | --- | --- |
| Discussion | | | | |
| Key results | 18 | Summarise key results with reference to study objectives | 9-10 | To the best of our knowledge, this is the first report about the predictive value of PNI on OS in NSCLC patients harboring EGFR mutations and following EGFR-TKIs. Our findings revealed that low pre-treatment PNI level correlated to worse OS for advanced NSCLC patients taking EGFR-TKIs. |
| Limitations | 19 | Discuss limitations of the study, taking into account sources of potential bias or imprecision. Discuss both direction and magnitude of any potential bias | 11, 12 | Our study was limited by its retrospective nature and relatively small number of patients from one single center. |
| Interpretation | 20 | Give a cautious overall interpretation of results considering objectives, limitations, multiplicity of analyses, results from similar studies, and other relevant evidence | 12 | However, as a whole, we demonstrated that the baseline PNI level correlated with the OS in patients harboring EGFR sensitive mutations and following EGFR-TKIs treatment. Besides, our study pointed out a potential intervention pointcut through which anti-inflammation and nutrition support may improve the prognosis. |
| Generalisability | 21 | Discuss the generalisability (external validity) of the study results | 12 | The results of our study suggest that the presence of a systemic inflammation and impaired nutritional status, as indicated by PNI, is a surrogate prognostic factor and the assessment of PNI could assist the prognosis identification for patients following EGFR-TKIs treatment and has implications for the routine monitoring and clinical intervention. |
| Other information | |  | | |
| Funding | 22 | Give the source of funding and the role of the funders for the present study and, if applicable, for the original study on which the present article is based | 12 | This study was supported by: National Natural Science Foundation of China (Grant No. 81372502, and 81201917), National High Technology Research and Development Program of China (Grant No. 2012AA02A502) and Innovative drug R&D center based on real-time high-throughput cell-based screening platform and large capacity compound library (Grant No. 2013ZX09401003-002).All the funders had no role in study design, data collection and analysis, decision to publish, or preparation of the manuscript. |

*Give information separately for cases and controls in case-control studies and, if applicable, for exposed and unexposed groups in cohort and cross-sectional studies.

**Note:** An Explanation and Elaboration article discusses each checklist item and gives methodological background and published examples of transparent reporting. The STROBE checklist is best used in conjunction with this article (freely available on the Web sites of PLoS Medicine at http://www.plosmedicine.org/, Annals of Internal Medicine at http://www.annals.org/, and Epidemiology at http://www.epidem.com/). Information on the STROBE Initiative is available at www.strobe-statement.org.
